# Immune Complex Formation Is Associated With Loss of Tolerance and an Antibody Response to Both Drug and Target

**DOI:** 10.3389/fimmu.2021.782788

**Published:** 2021-12-14

**Authors:** Mark A. Kroenke, Troy E. Barger, Jenny Hu, Mieke Jill Miller, Kevin Kalenian, Lidong He, Hailing Hsu, Yessenia Bartley, Vincent Fung-Sing Chow, Marcia Cristina Teixeira dos Santos, Barbara A. Sullivan, Laurence E. Cheng, Jane R. Parnes, Rupa Padaki, Scott Kuhns, Daniel T. Mytych

**Affiliations:** ^1^ Clinical Immunology, Translational Medicine, Amgen, Thousand Oaks, CA, United States; ^2^ Translational Safety & Bioanalytical Sciences, Amgen, Thousand Oaks, CA, United States; ^3^ Process Development, Attribute Sciences, Amgen, Thousand Oaks, CA, United States; ^4^ Inflammation Research, Amgen, Thousand Oaks, CA, United States; ^5^ Global Safety, Amgen, Thousand Oaks, CA, United States; ^6^ Clinical Pharmacology, Modeling and Simulation, Translational Medicine, Amgen, Thousand Oaks, CA, United States; ^7^ Clinical Biomarkers and Diagnostics, Translational Medicine, Amgen, Thousand Oaks, CA, United States; ^8^ Early Development, Translational Medicine, Amgen, Thousand Oaks, CA, United States

**Keywords:** immunogenicity, AMG 966, inflammatory bowel disease, anti-drug antibodies, TNFα, TL1A, tolerance, immune complexes

## Abstract

AMG 966 is a bi-specific, heteroimmunoglobulin molecule that binds both tumor necrosis factor alpha (TNFα) and TNF-like ligand 1A (TL1A). In a first-in-human clinical study in healthy volunteers, AMG 966 elicited anti-drug antibodies (ADA) in 53 of 54 subjects (98.1%), despite a paucity of T cell epitopes observed in T cell assays. ADA were neutralizing and bound to all domains of AMG 966. Development of ADA correlated with loss of exposure. *In vitro* studies demonstrated that at certain drug-to-target ratios, AMG 966 forms large immune complexes with TNFα and TL1A, partially restoring the ability of the aglycosylated Fc domain to bind FcγRIa and FcγRIIa, leading to the formation of ADA. In addition to ADA against AMG 966, antibodies to endogenous TNFα were also detected in the sera of subjects dosed with AMG 966. This suggests that the formation of immune complexes between a therapeutic and target can cause loss of tolerance and elicit an antibody response against the target.

## Introduction

Over the last two decades, protein-based therapeutics targeting tumor necrosis factor alpha (TNFα) have become a foundational therapy for the treatment of inflammatory disorders. These molecules have significantly improved patient outcomes in a number of diseases such as inflammatory bowel disease (IBD), which includes both Crohn’s disease (CD) and ulcerative colitis (UC). In CD specifically, protein-based therapeutics targeting TNFα have been shown to facilitate mucosal healing by endoscopy, reducing the need for surgical intervention (1, 2).

For CD patients, inducing and maintaining a state of deep remission is the treatment goal. However, even with an armamentarium of TNFα inhibitors for physicians to choose from, most patients do not achieve this goal. For patients being treated with adalimumab, for example, only 19% of patients with moderate to severe ileocolonic CD at 52 weeks demonstrated deep remission (3).

TNF-like ligand 1A (TL1A), encoded by the gene TNFSF15, has emerged as a cytokine potentially involved in IBD pathogenesis. Multiple genome-wide association studies have found single nucleotide polymorphisms in TNFSF15 that are associated with an increased risk of CD and UC (4, 5). Mice overexpressing TL1A developed spontaneous intestinal inflammation similar to human CD (6–8). Furthermore, dextran sulfate sodium or trinitrobenzenesulfonic acid-mediated colitis in mice both demonstrated a mitigation of disease when treated with a TL1A antagonist antibody, manifested as reduction in weight loss, mortality, and histologic score (6, 9).

Based on the human genetic data and the promise of TL1A inhibition in non-clinical models, as well as the established efficacy of TNFα inhibitors in IBD, Amgen designed AMG 966, a TNFα/TL1A bi-specific immunoglobulin therapeutic candidate. AMG 966 is a fully human, aglycosylated IgG1 antibody that binds both TNFα and TL1A. In order to bind both targets and still maintain the overall structure of a typical antibody, four unique heavy and light chains were paired by engineering amino acid substitutions known as charge pairs (10). Each heavy chain contains 4 and each light chain contains 2 complementary charge pair mutations. The charge pair mutations create a heteroimmunoglobulin molecule with two distinct antigen-binding fragments (Fabs). By virtue of the absence of an Fc glycan, AMG 966 is a stable effector functionless (SEFL) antibody, which lacks the ability to interact with Fcγ receptors (11).

To assess the safety and tolerability of AMG 966, a double-blind, placebo-controlled, first-in-human study was initiated in healthy subjects. The study included six single dose cohorts with doses ranging from 21 mg to 700 mg (either subcutaneous or intravenous administration), and three multiple dose cohorts with once every 2 weeks (Q2W) subcutaneous doses of 70, 210, and 420 mg. No significant safety concerns were noted, however, most subjects in the Q2W cohorts experienced an unexpected loss of exposure. Loss of exposure correlated with the onset of an anti-drug antibody (ADA) response which occurred in nearly all subjects.

Given that AMG 966 binds two trimeric targets and that each Fab domain is expected to bind one subunit of the trimer, we hypothesized that this robust anti-AMG 966 immune response was the consequence of immune complex formation. We demonstrate here that AMG 966 forms large immune complexes with target which partially restores the ability of the SEFL Fc to bind FcγR, providing a mechanism by which these complexes drive an antibody response to both AMG 966 and endogenous TNFα.

## Materials and Methods

### Study Design

Study 20160316 was a randomized, double-blind, placebo-controlled study to evaluate the safety, tolerability, and pharmacokinetics of AMG 966 in healthy subjects. The study was conducted under United States Food and Drug Administration investigational new drug application 131513. Informed consent was obtained from all subjects before participation.

### *In Vitro* T Cell Assays

Donors were recruited at phase 1 clinical trial units and selected to represent the global frequency of HLA-DRB1 alleles. For the PBMC assay, cells were seeded in a 96 well plate at a density of 2.5x10^5^ cells per well. Test proteins were added at a concentration of 300 nM, with each condition carried out with 8 replicates. On day 7, CD3^+^ CD4^+^ Edu^+^ cells were measured by flow cytometry. For the DC:T assay, monocytes were isolated from PBMCs through positive selection, and differentiated into immature dendritic cells using GM-CSF and IL-4. Immature dendritic cells were loaded with test proteins and matured using TNFα and IL-1β to yield mature dendritic cells. Autologous CD4 T cells were isolated from PBMC using negative selection and co-cultured with the mature dendritic cells for 6 days, with each condition carried out in 6 replicates. The stimulation index was calculated by dividing the test condition by the media alone control (baseline).

### Anti-Drug Antibody Assay

Anti-AMG 966 antibodies were measured using a validated, electrochemiluminescence-based bridging assay with both screening and confirmatory components. Prior to analysis, samples were treated with 300 mM acetic acid to enable antibody-drug complex dissociation. Then, acid treated samples were neutralized and incubated in a mixture of biotinylated-AMG 966, ruthenylated-AMG 966, and etanercept. ADA present in serum samples form a bridge between the two AMG 966 conjugates, while etanercept blocks interference from soluble TNFα which may be present in serum samples. The formed antibody complex was captured on a blocked streptavidin plate, washed, and analyzed on a plate reader where signal was produced from an electrically induced oxidation-reduction reaction involving ruthenium and tripropylamine. Samples with a signal to noise ratio higher than the assay cut point in the screening assay were treated with excess AMG 966 in the confirmatory assay to assess specificity. Percent depletion was calculated by subtracting the S/N from the treated specimen from the S/N of the untreated specimen and dividing by the untreated specimen S/N value. The screening and confirmatory assay cut points were calculated from 51 healthy donor serum samples in accordance with regulatory guidance. The combined sensitivity of the screening and confirmatory components was 4.6 ng/mL based on a rabbit polyclonal positive control antibody. At 100 ng/mL of anti-AMG 966 antibody, the assay tolerated at least 10 μg/mL of excess AMG 966.

### Pharmacokinetic Assay

AMG 966 was measured in serum using a validated sandwich immunoassay. The assay utilized an anti-idiotype monoclonal antibody against the TL1A Fab domain for capture, and a biotin conjugated anti-idiotype monoclonal antibody against the TNFα Fab domain for detection. The assay range was 50 to 5,000 ng/mL.

### Neutralizing Antibody Assay

A multiplex, competitive target binding assay was utilized for the simultaneous detection of neutralizing antibodies against both domains of AMG 966. In this assay, biotinylated TNFα and TL1A were coupled to U-PLEX linkers (Meso Scale Diagnostics, Rockville, MD). The coupled U-PLEX linkers were then self-assembled onto respective spots on the U-PLEX plates. Ruthenylated AMG 966 (Ru-AMG 966) was first incubated with sample and then added to the U-PLEX plate. Neutralizing antibodies against either domain of AMG 966 competed with biotinylated TNFα and/or TL1A for binding to Ru-AMG 966, resulting in a reduced electrochemiluminescent (ECL) signal. To confirm that a sample contained anti-AMG 966 specific neutralizing antibodies, an excess amount of AMG 966 was added exogenously to the sample at 3 different concentrations, resulting in a bell shape ECL response. In this assay format, a majority of excess AMG 966 at lower concentrations will competitively bind to anti-AMG 966 antibodies and allow Ru-AMG 966 to re-bind to the TNFα and/or TL1A, thus resulting in an increase in ECL signal in the assay. As the concentration of added AMG 966 is increased, eventually the exogenous AMG 966 will directly compete with Ru-AMG 966 for binding to TNFα and/or TL1A, causing signal to decrease. A sample was considered positive for neutralizing antibodies if a 1.5-fold increase in ECL signal was observed at any of the 3 concentrations of exogenous AMG 966. Assay sensitivity for anti-TNFα domain and anti-TL1A domain of AMG 966 was 150 ng/mL and 200 ng/mL, respectively, based on a rabbit polyclonal anti-AMG 966 positive control antibody.

### SEC-MALS

Immune complexes of drug and target were created by incubating AMG 966 with TNFα and/or TL-1A, along with other control molecules, in different molar ratios at 4°C for approximately 16 hours. Size exclusion chromatography with in-line multi-angle light scattering (SEC-MALS) was used to characterize the samples.

SEC-MALS was performed using an Infinity II UHPLC pump and autosampler (Agilent, Santa Clara, CA) connected in series to an Acquity UPLC Protein SEC Guard Column (450Å, 2.5 µm, 4.6 mm X 30 mm, Waters, Milford, MA) connected to an Acquity UPLC BEH450 SEC column (450Å, 2.5 µm, 4.6 mm X 300 mm, Waters, Milford, MA). The effluent of the SEC column flowed through an inline UV/Vis detector (Agilent), microDawn™ multi-angle light scattering detector (Wyatt Technologies, Santa Barbara, CA) and a microOptilab refractometer (Wyatt Technologies, Santa Barbara, CA). A mobile phase composed of 20 mM Sodium Phosphate, 250 mM NaCl, 5% Ethanol, pH 7.0 was used at a flow rate of 0.25 mL/min. with an overall run time of 20 minutes per sample. Approximately 30 µg of protein was injected onto the SEC column. SEC-MALS data were analyzed using ASTRA software (Wyatt Technologies) to determine the molar mass (Mw) across the SEC chromatogram.

### FcγR Competitive Binding Assay

The relative FcγR binding levels were determined by using an Amplified Luminescent Proximity Homogenous (AlphaLISA). The assay is based on a luminescent bead-based system available from Perkin Elmer (Waltham, MA). The details of the chemistry behind the reaction are available from vendor product literature, but briefly, the assay utilizes two bead types, a glutathione-coated acceptor bead which binds recombinant human FcγR-Glutathione-s-transferase (FcγR-GST), and a streptavidin donor bead which contains a photosensitizer and binds to biotinylated human IgG1. Binding of FcγR-GST and biotinylated human IgG1 brings the acceptor and donor beads into close proximity. When laser light is applied to this complex, ambient oxygen is converted to singlet oxygen by the donor bead. If the beads are in proximity, an energy transfer to the acceptor bead occurs, resulting in light production (luminescence), which is measured by a plate reader equipped for AlphaLISA signal detection. When human IgG is present at sufficient concentrations to inhibit the binding of FcγR-GST to the biotinylated human IgG1, a dose-dependent decrease in emission is observed.

Glutathione-coated acceptor beads were coated with recombinant human Fc gamma Receptor-GST-H6 diluted to the required final concentration in AlphaLISA™ buffer (PerkinElmer). This mixture was incubated in the dark for 2 to 4 hours at room temperature. IgG1 positive control and AMG 966 sample material were serially diluted in AlphaLISA buffer and transferred to a mixing plate. Biotinylated IgG1 competitor was added to all wells of the mixing plate from low to high concentration, mixed well and then transferred to each appropriate well of the three replicate assay plates. Fcγ Receptor coated acceptor beads were then added to each well of the assay plates, and the plates were incubated in the dark for 22 to 26 hours at room temperature. Following incubation, streptavidin donor beads were added to each well of the assay plates and again incubated in the dark for 2 to 6 hours at room temperature. Assay plates were then read on the Perkin Elmer (Waltham, MA) at 680 nm excitation and 570 nm emission. Each data point of the dilution curve was run in triplicate across three assay plates.

Data analysis was performed in accordance with bioassay guidance found in the United States Pharmacopeia (USP) chapters 1030 and 1032. Briefly, data were fitted to the mean emission values using a 4-parameter curve fit using SoftMax Pro software. The data analysis was conducted for each three-plate setup using a 4-parameters, non-weighted model. The curve equation is as follows:


$$\text{y}=\text{d}+\{(\text{a}-\text{d})/[1+{\left(\text{x}/\text{c}\right)}^{\text{b}}]\}$$

Where coefficient **a** controls the location of the lower asymptote, coefficient **d** controls the location of the upper asymptote, coefficient **b** controls the rate of approach to the asymptotes and the rate of transition, and coefficient **c** controls the location of the transition. Results are reported as percent relative binding as calculated by the IC50 reference standard/IC50 sample.

Binding to FcγRIA, FcγRIIA, and FcγRIIIA (158V) was evaluated using the above method. AMG 966 sample material, recombinant human TNFα, and recombinant human TL1A were mixed at various molar ratios one day prior to assay execution and incubated at 4°C overnight in order to allow immune complexes to form. AMG 966: TNFα: TL1A ratios used across all assays were 1:1:1, 4:1:1, 9:1:1, as well as AMG 966 alone. A 100:1:1 ratio was also included in the FcγRIA assay.

In the FcγRIA assay, IgG1 positive control was tested at a concentration range of 400 nM to 0.062 nM and sample material was tested at a concentration range of 1800 nM to 14.063 nM. In the FcγRIIA assay, both IgG1 positive control and sample material were tested at a concentration range of 1800 nM to 0.823 nM. In the FcγRIIIA (158V) assay, IgG1 positive control was tested at a concentration range of 1600 nM to 0.732 nM and sample material was tested at a concentration range of 1800 nM to 0.823 nM.

### TNFα and TL1A Measurements

Approximately 5 mL of blood for biomarker analysis were obtained at the study site. Blood samples were collected into EDTA tubes. Plasma was separated by centrifugation (1500 x g for 15 minutes at room temperature) within 1 h of collection and stored frozen at −70 °C. TNFα and TL1A levels were measured in duplicates by single molecule array (Simoa) technique (Quanterix, Lexington, Massachusetts). Briefly, frozen samples were thawed on ice and centrifuged at 20,000 x g for 3 minutes to pellet any debris. Samples were transferred to a 96 well plate, diluted 1:4 with appropriate sample diluent and tested in series on a single Simoa HD-1 Analyzer. Calibration curves were made fresh from frozen aliquots. Controls, defined as large volume calibrators were treated like samples. Scientists were blinded to subjects’ assignment.

### Surface Plasmon Resonance Assay

Clinical serum samples were analyzed in a SPR immunoassay qualified to detect anti-TNFα or anti-TL1A antibodies. In brief, recombinant human TNFα or TL1A was immobilized onto a biosensor chip flow cell. Then, samples were evaluated in a two-part series. Part one included passing diluted samples over the immobilized surface to allow anti-TNFα or anti-TL1A antibody specific binding followed by signal enhancement with anti-human IgG. In part two, again serum antibodies bound to the immobilized protein and signal enhancement with mAb 1A3 (specific to AMG 966) was used in place of anti-human IgG. Assay signal was adjusted to account for the assay background mean which resulted in net response units (RU). Antibody binding with enhanced reactivity only to anti-human IgG confirmed presence of anti-TNFα or anti-TL1A antibodies. Otherwise, enhanced reactivity to mAb 1A3 indicated presence of soluble drug in samples. Serum spiked with soluble AMG 966 drug served as the assay positive control and unspiked serum was used for the negative control. Assay sensitivity for detection of anti-TNFα was 63 ng/mL and detection of anti-TL1A was 60 ng/mL.

## Results

### Preclinical Assessment of Immunogenic Risk for AMG 966

In order to assess the immunogenic risk of AMG 966 and the charge pair mutations (**Supplementary Figure 1**), an in silico analysis of MHC class II binding was performed using the Immune Epitope Database (IEDB) tool. Using a percentile rank threshold of ≤1%, IEDB predicted that eight new agretopes, all in the TNFα binding domain, would be formed as a consequence of the 12 charge pair mutations (**Supplementary Table 1**).

**Figure 1:**
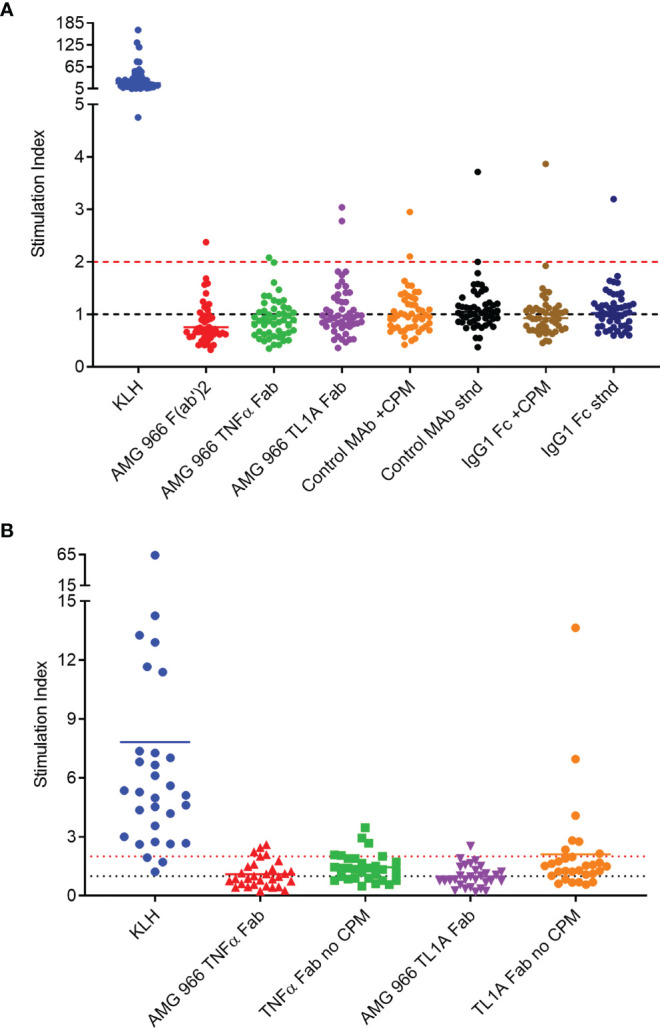
*In vitro* T cell assays did not reveal sequence-based risk of immunogenicity for AMG 966. T cell assays were performed with naïve donors representative of global HLA allele frequencies. Results are shown as stimulation index, or test protein divided by the baseline condition (media alone). **(A)** PBMC from 50 donors were stimulated with each test protein for 6 days prior to assessment of CD4 T cell proliferation by flow cytometry. A control monoclonal antibody and an IgG1 Fc domain were tested both with charge pair mutations (CPM) and without (stnd). **(B)** Monocytes from 30 PBMC donors were differentiated into dendritic cells, loaded with test protein, and matured. Autologous CD4 T cells were isolated and co-cultured with mature dendritic cells presenting test protein agretopes for 6 days prior to assessment of CD4 T cell proliferation by flow cytometry. Fab domains were tested with and without CPM.

It is well-established, however, that in silico prediction of class II binding has a high false positive rate (12–14). In order to further assess the immunogenic risk of AMG 966, a set of T cell assays was performed utilizing AMG 966 domains both with and without the charge pair mutations. First, a conventional T cell assay with peripheral blood mononuclear cells (PBMCs) was performed. This assay involves stimulation of PBMCs with test protein for 6 days, followed by an overnight assessment of CD4 T cell proliferation by EdU incorporation. Naïve donors were selected such that they represented the global frequency of HLA DRB1 alleles. All donors demonstrated a robust response to keyhole limpet hemocyanin (KLH) which was used as a positive control. The T cell response to the AMG 966 F(ab’)_2_, the AMG 966 TNFα Fab, and the AMG 966 TL1A Fab was limited, with 1 or 2 donors out of 50 showing a stimulation index >2 (**Figure 1A**). To further assess potential T cell responses elicited by the charge pair mutations, a control heteroimmunoglobulin monoclonal antibody with the same engineered charge pair mutations as AMG 966 was tested alongside the standard version of the same control antibody. The presence of the charge pair mutations had no impact on the T cell response. The same outcome was observed when only the Fc domain was assessed, with and without the charge pair mutations.

In order to rule out the possibility that neutralization of TNFα and/or TL1A had some impact on the outcome of the T cell assay, a DC:T cell assay was performed. In this assay format, the antigen presenting cells are loaded with the test protein in the absence of T cells, washed, and then autologous T cells are added back at a later time point, ensuring that the function of the test protein has no impact on the outcome of the assay. Consistent with the PBMC-based assay, the charge pair mutations did not impact the T cell response in the DC:T assay (**Figure 1B**). Together, these preclinical assessments indicated minimal to no risk of immunogenicity in clinical studies.

### Clinical Immunogenicity Assessment in the AMG 966 First-In-Human Study

**Figure 2:**
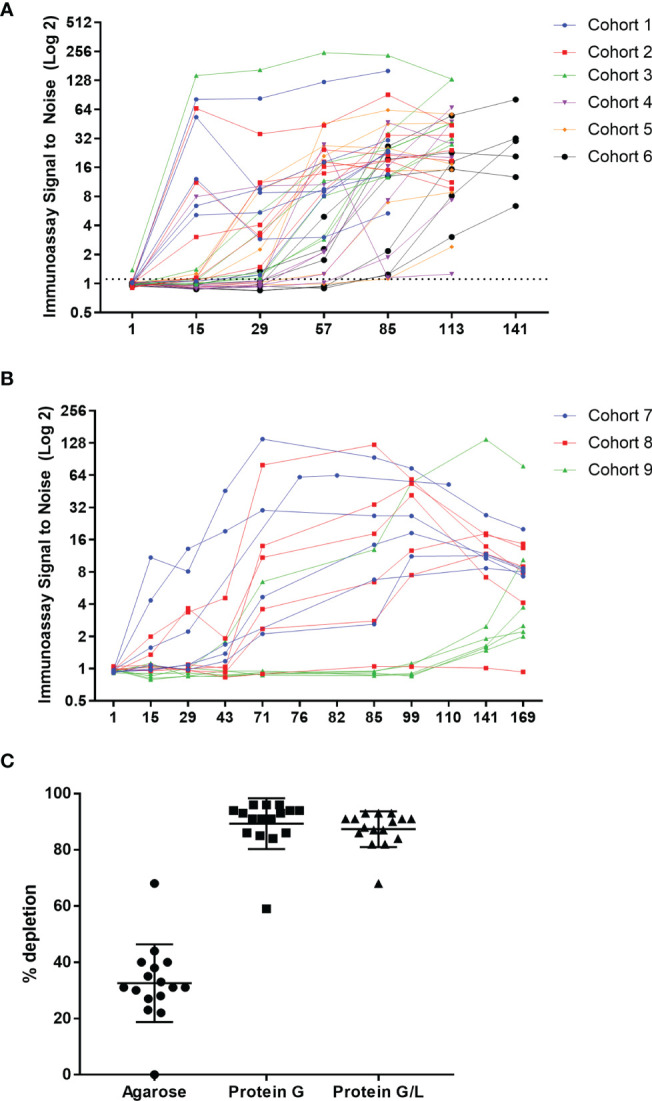
AMG 966 was highly immunogenic in all cohorts. **(A)** The magnitude of the anti-AMG 966 antibody response (signal to noise ratio) is shown over time. Every subject in the SAD cohorts developed anti-AMG 966 antibodies. The study duration varied with dose, and the end of study antibody sample was taken on day 85 for cohort 1, day 113 for cohorts 2-5, and day 141 for cohort 6. **(B)** S/N for all AMG 966 dosed subjects from the MAD cohorts is shown. 17 of 18 subjects developed anti-AMG 966 antibodies by end of study, day 169. Subjects in these cohorts were dosed Q2W with the last dose administered on day 71. **(C)** Pre-treatment of serum samples with protein G or protein G/L beads depleted signal in the immunoassay to background levels, confirming that signal in the immunoassay is antibody derived.

The first-in-human study was randomized and placebo controlled, with six single ascending dose (SAD; **Supplementary Figure 2A**) and three multiple ascending dose (MAD; **Supplementary Figure 2B**) cohorts. The single ascending dose portion of the study was designed to assess the safety and tolerability of AMG 966 at each dose level. Based on acceptable safety and tolerability profile observed after single doses of AMG 966, the study was amended to further explore multiple ascending doses of AMG 966. Serum for ADA testing was collected at baseline and at regular intervals after AMG 966 administration. ADA were assessed using a validated bridging assay. A rapid anti-AMG 966 antibody response was observed, with 5 of 6 dosed subjects in cohort 1 testing positive for ADA at day 15, the first post-dose time point (**Figure 2A**). By the end of the study, every subject in the SAD cohorts had tested positive for ADA at one or more time points. In the MAD cohorts, all subjects but one individual in cohort 8 (210 mg) were positive for ADA at one or more timepoints (**Figure 2B**). In general, the magnitude of the ADA response, measured as signal to noise ratio (S/N), increased over time. It should be noted, however, that the S/N ratio was likely suppressed due to interference from circulating soluble drug in serum samples, particularly in higher dose cohorts. In total, the incidence of ADA in the FIH study was higher than expected based on the immunogenicity risk assessment, and 53 of 54 subjects (98.1%) developed anti-AMG 966 antibodies.

Bridging immunoassays are susceptible to false positive results if the drug target is multimeric and present in serum samples (15). In order to rule out the possibility that an increase in soluble trimeric TNFα or TL1A was causing a false positive in the ADA assay, each sample was pre-treated with a protein G coated bead or a bead coated with protein G and protein L together to deplete immunoglobulins (with an agarose “blank” bead used as a control), and then re-tested in the ADA assay. Pre-treatment with either protein G or protein G/L caused significant depletion of signal in the ADA assay, confirming that the signal in the assay is a result of anti-AMG 966 antibodies (**Figure 2C**). Furthermore, because protein L, but not protein G, can bind IgM and IgA, the similarity between protein G and protein G/L depletion indicates the ADA are primarily class switched IgG.

### No Impact of Immunogenicity on Safety

Despite the high incidence of anti-AMG 966 antibodies, there were no ADA-related adverse events reported. The most common treatment-emergent adverse events were injection site bruising and ecchymosis for the SAD and MAD portions of the study, respectively (**Table 1**). No serious adverse events were reported.

**Table 1:** Treatment-emergent adverse events in AMG 966 treated subjects.

	SAD N=36	MAD N=18
Ecchymosis	0	5
Injection site bruising	4	0
Erythema	1	4
Leukocytosis	2	0
Headache	1	2
Abdominal discomfort	0	1
Abdominal distension	0	1
Acne	0	1
Alanine aminotransferase increased	0	1
Eye irritation	1	0
Injection site haemorrhage	0	1
Injection site swelling	1	0
Laryngitis	0	1
Pruritus	0	1
Pyrexia	0	1
Rash	1	1
Upper respiratory track infection	1	0
Urticaria	0	1

### Impact of Immunogenicity on AMG 966 Exposure

**Figure 3:**
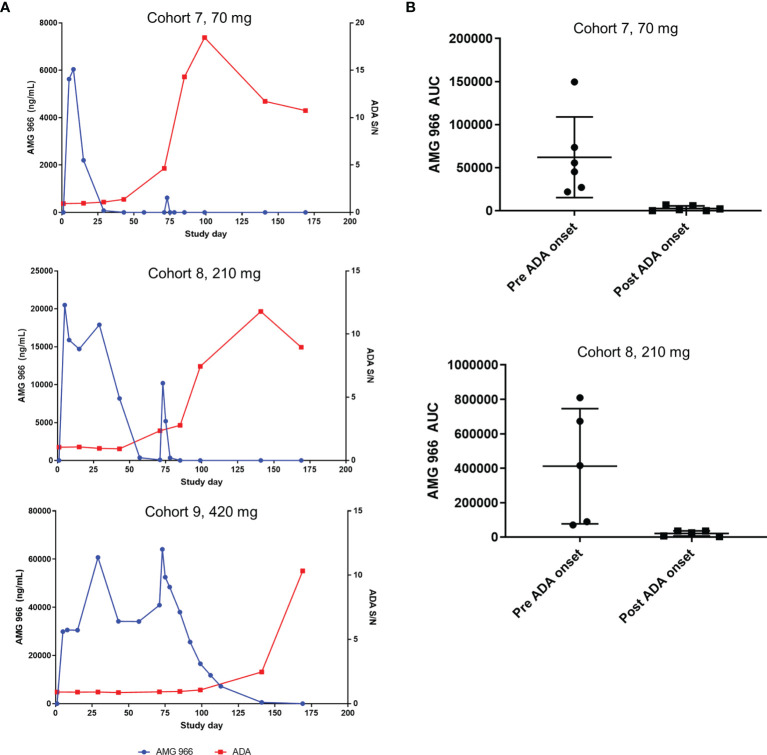
Anti-AMG 966 antibodies impacted exposure in MAD cohorts. **(A)** AMG 966 concentration is plotted together with the magnitude of the anti-AMG 966 antibody response, shown as signal to noise ratio. Representative subjects are shown from each MAD cohort. Subjects were dosed Q2W with the last dose administered on day 71. **(B)** AMG 966 area under the curve (AUC) is shown pre and post ADA onset in cohorts 7 and 8. ADA onset was defined as the first study timepoint with an ADA positive result. For cohort 9, all subjects maintained exposure throughout the dosing phase, and ADA onset was too late to assess impact on AUC.

In the SAD cohorts, exposure was within a 2-fold range of what models predicted based on non-clinical data. However, assessment of the impact of immunogenicity in a SAD cohort is difficult, since most serum AMG 966 has been cleared prior to the onset of a humoral immune response. In the MAD cohorts 7 and 8, clear impact of ADA on the pharmacokinetics (PK) of AMG 966 was observed. In general, an increase in ADA magnitude correlated with loss of AMG 966 exposure on a per subject basis (**Figure 3A**). The mean area under the curve (AUC) during the dosing phase (defined as day 1 through day 85) decreased significantly post-ADA onset for cohorts 7 and 8 (**Figure 3B**). All subjects in cohort 9 maintained exposure throughout the dosing phase but became ADA positive by the end of the study. It was not clear if maintenance of exposure in cohort 9 was due to the 420 mg dose mitigating antibody formation (i.e. high dose tolerance), or if the higher concentrations of AMG 966 in sera competed with the labeled ADA assay reagents, thereby delaying detection of ADA. Across all cohorts, ADA onset tended to be more delayed at higher doses, suggesting the latter possibility.

### Characterization of the Anti-AMG 966 Antibody Response

The AMG 966 PK assay utilizes two anti-idiotype antibodies and only measures free AMG 966 that is not bound to either target. As a result, there was a possibility that a significant amount of bioactive drug was present that was not measurable (i.e. AMG 966 with the TL1A Fab free, but TNFα Fab bound to ADA or vice versa). In order to further understand the nature of the anti-AMG 966 antibody response, a neutralizing antibody assay was developed to assess the ability of anti-AMG 966 antibodies to interfere with binding of AMG 966 to targets.

Briefly, TL1A and TNFα were coated on distinct spots on a UPlex MSD plate and signal was measured using ruthenylated AMG 966. Neutralizing antibodies to AMG 966 result in loss of signal in this assay. Samples from cohort 1 and cohort 7 were tested as representative samples from the SAD and MAD cohorts, respectively. By the end of the study, all ADA positive subjects were positive for neutralizing antibodies to the TL1A binding domain, and 7 of 12 were positive for neutralizing antibodies to the TNFα binding domain, suggesting that the observed loss of exposure was primarily due to neutralizing anti-AMG 966 antibodies that competed for binding with the anti-idiotype antibodies used in the PK assay (**Table 2**).

**Table 2:** Incidence of anti-AMG 966 neutralizing antibodies to the TNFα and TL1A binding domains.

		TNFα	TL1A
Cohort 1	Day 57	1/6	0/6
	Day 85/EOS	2/6	6/6
Cohort 7	Day 85	0/6	3/6
	Day 169/EOS*	5/6	6/6

*One cohort 7 subject withdrew from the study early and had an antibody sample taken at day 110 instead of day 169.

**Figure 4:**
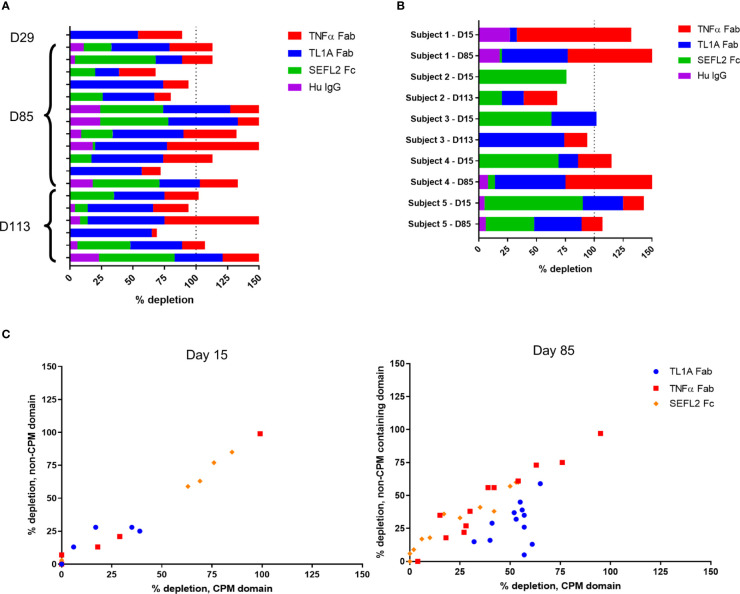
Anti-AMG 966 antibodies bound to all domains of AMG 966 and became specific for TL1A charge pair mutations over time. Domain characterization was performed by pre-treating serum samples with various domains of AMG 966 and then re-testing in the ADA assay to determine the extent to which assay signal was depleted. Percent depletion was calculated by dividing the difference between treated and untreated samples by the untreated signal. **(A)** Antibody positive samples from 19 subjects from cohorts 1-4 were assessed for binding to each domain of AMG 966 or bulk human IgG negative control. The timepoint for each sample is indicated on the y-axis. **(B)** Paired antibody positive samples from the same subject were analyzed at day 15 and day 85/113 to explore how the specificity of the antibody response changes over time. Data shown represent 2 paired samples from cohort 1, 2 pairs from cohort 2, and 1 pair from cohort 3. **(C)** Antibody positive samples were pre-treated with either a wild-type domain or a domain containing the charge pair mutations to assess the extent to which antibody binding was dependent on charge pair mutations. Data shown were derived from 19 subjects from cohorts 1-4.

To further characterize the anti-AMG 966 immune response, domain characterization was performed by pre-treating serum samples with various domains or modified versions of AMG 966, then running those samples in the ADA assay and comparing the signal relative to the untreated sample. Using this approach, a depletion of signal to a particular domain would indicate that a portion of the anti-AMG 966 antibody response was directed towards that domain. All subjects from cohorts 1 through 3, and 1 subject from cohort 4 were evaluated at a variety of time points. While several subjects showed binding to the TNFα and TL1A Fab domains alone, the majority of subjects showed binding to all 3 domains of the molecule, with minimal binding to the human IgG negative control (**Figure 4A**). In some cases, depletion exceeded 100%, presumably because some antibody clones are specific for epitopes that are present in more than one domain. For a subset of 5 subjects, matched pairs of ADA positive samples were analyzed for day 15 and a late antibody timepoint, either day 85 or day 113. Interestingly, in 4 of 5 subjects, the early antibody response was primarily directed towards the Fc domain, but over time, shifted to primarily recognize the Fab domains (**Figure 4B**). The importance of the charge pair mutations for antibody binding was assessed by depleting samples with individual protein domains with and without the charge pair mutations. At day 15, ADA binding was independent of the charge pair mutations, as indicated by equivalent depletion for domains with and without charge pair mutations. By day 85, however, many subjects had developed a preference for binding the charge pair mutations in the TL1A Fab, and some lost nearly all ability to bind to the non-charge pair mutation containing domain (**Figure 4C**). Domain characterization was not performed on the MAD cohorts since in most subjects, there was not adequate signal to perform domain characterization until late in the study, at which point affinity maturation may have already occurred.

### AMG 966 Forms Large Immune Complexes With Target *In Vitro* and Restores FcγR Binding

**Figure 5:**
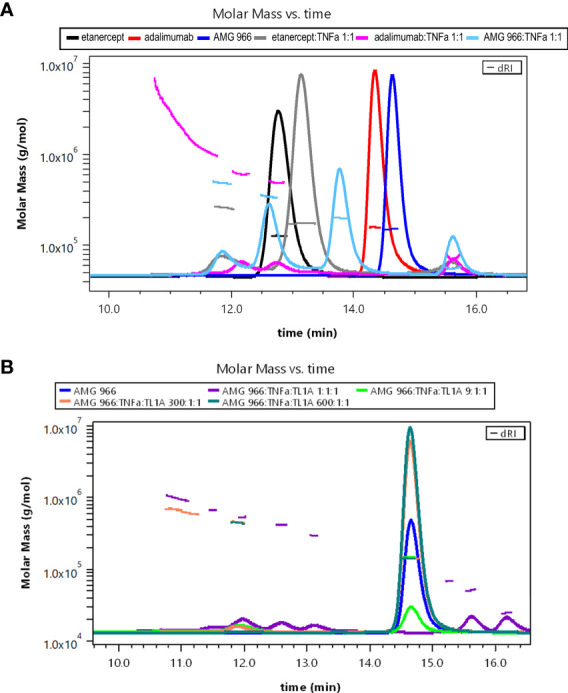
AMG 966 forms large complexes with TNFα and TL1A *in vitro*. SEC-MALS analysis was performed after overnight incubation of various therapeutic proteins and trimeric target at different ratios. **(A)** Etanercept, adalimumab, and AMG 966 were analyzed either alone, or after being mixed in a 1:1 ratio with TNFα. **(B)** AMG 966 was combined with TNFα and TL1A at various ratios and analyzed.

Bivalent molecules, such as monoclonal antibodies, that bind multivalent targets can form large immune complexes (16, 17). We hypothesized, given the ability of AMG 966 to bind two trimeric targets, that immune complex formation was driving the immune response to AMG 966. In order to test this hypothesis, immune complexes were created *in vitro* using different ratios of drug to target and analyzed by SEC-MALS. Etanercept and adalimumab were used as controls, and consistent with published data, showed that each molecule of etanercept binds one TNFα trimer, while adalimumab formed much larger complexes with TNFα. When mixed with TNFα in a 1:1 ratio, AMG 966 resulted in complexes that were between etanercept and adalimumab complexes in terms of size (**Figure 5A**). Introducing TL1A into the mixture of AMG 966 and TNFα enhanced complex formation, with larger complexes forming at lower ratios of drug to target (**Figure 5B**). Despite having the ability to bind two distinct trimeric targets, AMG 966 did not create larger immune complexes than adalimumab.

**Figure 6:**
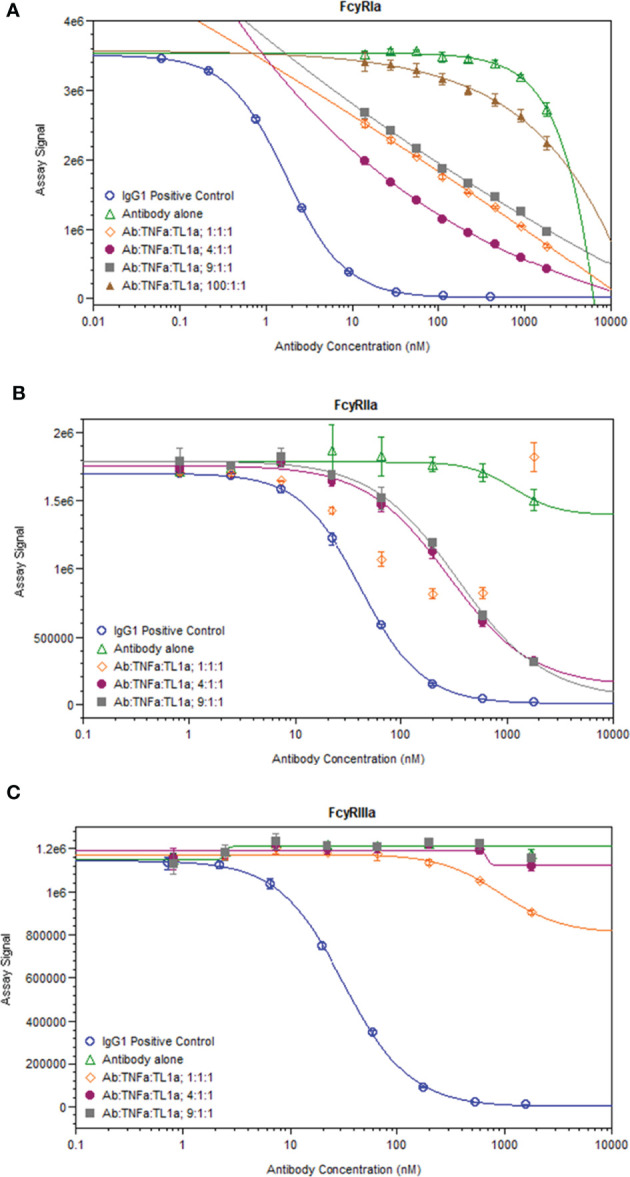
AMG 966 complex formation with targets restored binding to FcγRIa and FcγRIIa. Competitive binding assays were utilized to assess the impact of AMG 966 complex formation with targets on FcγR binding. An IgG1 positive control antibody was used in each assay and compared to AMG 966 alone and various ratios of AMG 966/target complex for ability to bind FcγRIa **(A)**, FcγRIIa **(B)**, or FcγRIIIa **(C)**.

While immune complexes are known to be immunogenic, immune complex-mediated immunogenicity is typically driven by mechanisms that are lacking from the aglycosylated SEFL Fc domain of AMG 966, such as FcγR binding (11). We investigated whether complex formation restores these effector functions of the AMG 966 Fc domain by significantly increasing avidity, which was plausible based on studies of how protein aggregation impacts FcγR binding (18–20) and published data exploring FcγR binding properties of other TNFα inhibitors (21). In order to test this, we formed AMG 966 immune complexes with targets *in vitro* and assessed binding to FcγRIa, FcγRIIa, and FcγRIIIa compared to AMG 966 alone in a competitive AlphaLISA binding assay. At low drug to target ratios, the AMG 966:target complexes exhibited enhanced FcγRIa and FcγRIIa binding, which diminished as the ratio increased (**Figures 6A, B**), consistent with the immune complex formation observed by SEC-MALS. There was minimal impact of complex formation on FcγRIIIa binding (**Figure 6C**). Relative potency at each ratio is shown in **Supplementary Table 2**.

### AMG 966 Causes an Antibody-Associated Increase in Serum TNFα

**Figure 7:**
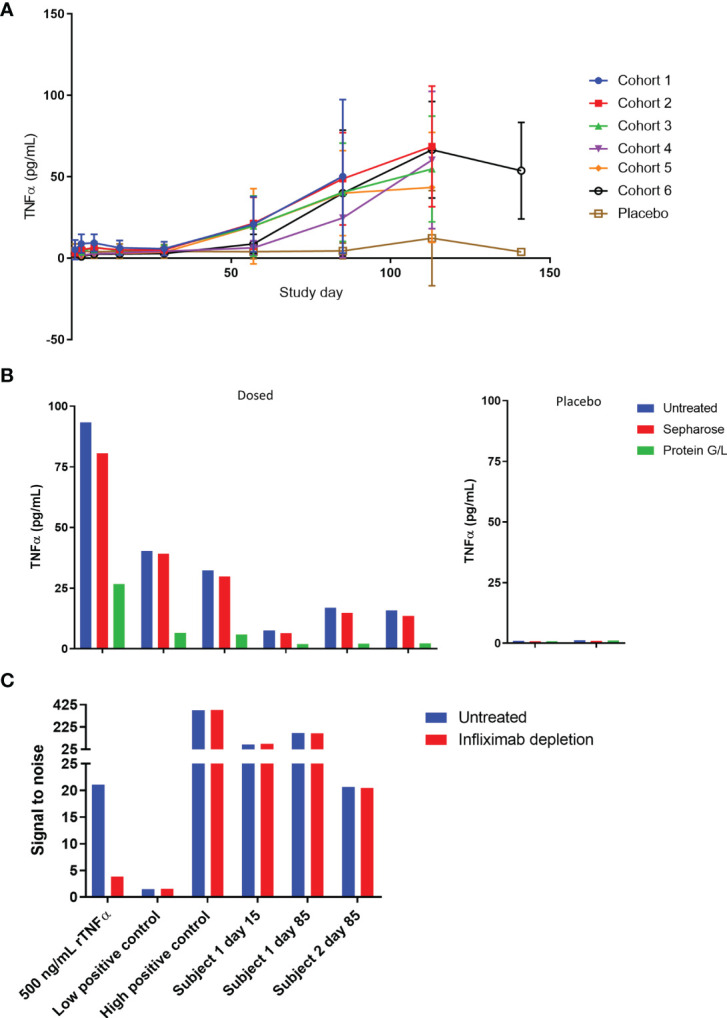
TNFα is bound to immunoglobulin and accumulates in serum over time. **(A)** Serum TNFα was measured for all subjects in the SAD cohorts throughout the course of the study. **(B)** Serum samples from cohort 1 at day 85 were pre-treated with protein G/L beads or blank sepharose beads and tested alongside untreated samples in the TNFα assay to assess whether TNFα was bound to immunoglobulin. **(C)** Serum samples were pre-treated with excess infliximab and re-tested in the ADA assay to confirm that the assay was not yielding false positive results due to 1:1 complexes of TNFα and endogenous anti-TNFα antibody. A high concentration of TNFα known to give a false positive was used as a control and tested alongside low and high positive controls (polyclonal anti-AMG 966 antibody).

As part of the pharmacodynamic assessments for the first-in-human study, serum TNFα and TL1A levels were measured. Instead of decreasing as expected, TNFα signal in SAD cohorts gradually increased over time (**Figure 7A**) while placebo TNFα levels remained steady. A similar phenomenon was not observed in the TL1A assay, and TL1A levels gravitated towards baseline as the study progressed (**Supplementary Figure 3**). The TNFα assay is a bead-based immunoassay which utilizes a monoclonal capture reagent and a polyclonal detector that can detect TNFα trimer with only 1 free subunit. Based on this, we hypothesized that AMG 966 complexed with target led to a break in tolerance and production of antibodies to both the drug and to TNFα. These endogenous TNFα antibodies bind to 1 or 2 subunits of the TNFα trimer, effectively neutralizing TNFα function by preventing the trimer from binding its receptor. At the same time, these TNFα-antibody complexes extend the half-life of TNFα, leading to an increase in TNFα assay signal over time, but none of the adverse events that would normally be associated with such high levels of TNFα. In order to test this hypothesis, we pre-treated serum samples with a protein G/L bead or a sepharose control bead and tested the samples again in the TNFα immunoassay. Consistent with antibody binding to TNFα, the protein G/L, but not sepharose control, depleted the signal in the TNFα assay (**Figure 7B**).

These data also raised the possibility that the ADA assay was not detecting true ADA, but 1:1 complexes of endogenous anti-TNFα antibodies and trimeric TNFα, since partially bound TNFα trimer could still theoretically bridge two molecules of AMG 966 in the antibody assay, and the etanercept spiked into the assay buffer to address this issue would not be able to neutralize partially bound TNFα. In order to rule this out, a subset of ADA samples was pre-treated with infliximab and retested in the ADA assay. If the assay were measuring TNFα bound to endogenous anti-TNFα antibodies then infliximab, which binds to individual TNFα subunits, should deplete the assay signal (16, 22). As a positive control, a serum sample spiked with a high concentration of TNFα known to give a false positive in the antibody assay was included to confirm that infliximab could deplete TNFα-mediated signal. Infliximab was able to deplete the signal from the recombinant TNFα spiked sample but had no impact on the assay signal in any of the clinical samples, indicating that the antibody assay was measuring bona fide anti-AMG 966 antibodies (**Figure 7C**).

### AMG 966 Exposure Is Associated With an Endogenous Anti-TNFα Antibody Response

In order to test the hypothesis that AMG 966 immune complexes with target were leading to an endogenous antibody response to TNFα, a surface plasmon resonance (SPR) assay was developed. The assay included both a TL1A and a TNFα flow cell surface for antibody capture. Binding of immunoglobulin to the flow cell surface protein was confirmed with a polyclonal, anti-human IgG antibody. While samples for this assay were chosen to have undetectable levels of AMG 966, an anti-AMG 966 monoclonal antibody was also used in parallel as a detection reagent to ensure binding signal in the assay was not due to AMG 966. This anti-AMG 966 antibody, termed 1A3, is highly specific for AMG 966 and does not recognize endogenous immunoglobulin.

**Figure 8:**
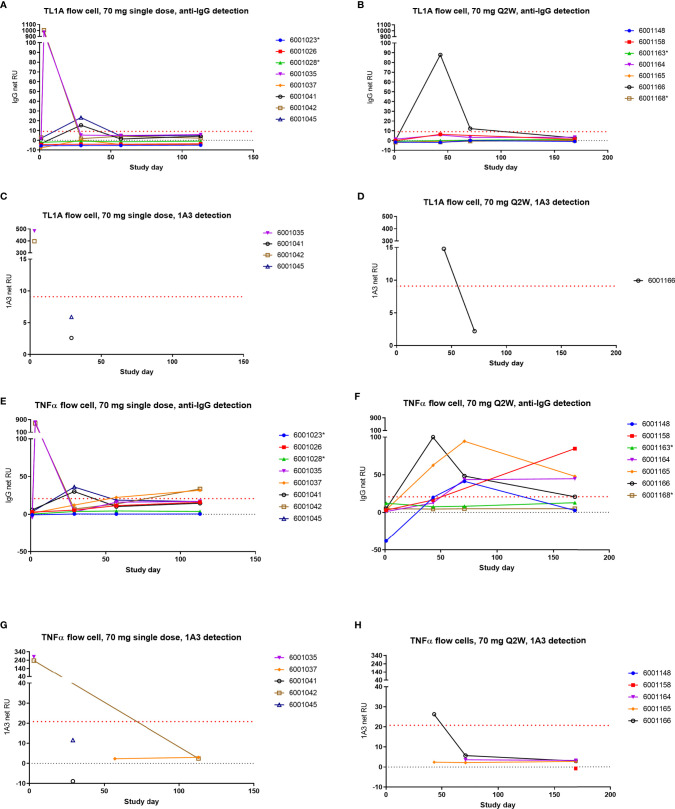
AMG 966 elicited endogenous antibodies to TNFα but not TL1A. Subjects from cohorts 2 and 7 were tested for endogenous antibodies to TNFα and TL1A using an SPR approach. One AMG 966-treated subject from cohort 7 who withdrew from the study early was not included. Samples with reactivity for TL1A or TNFα based on anti-human IgG detection were further tested using the AMG 966-specific 1A3 reagent for detection. Asterisks indicate subjects in the placebo group. Cohort 2 **(A)** and cohort 7 **(B)** were assessed for TL1A binding antibodies and reactive samples were further tested for 1A3 binding **(C, D)**. Cohort 2 **(E)** and cohort 7 **(F)** were also tested for TNFα binding antibodies and reactive samples were further tested for 1A3 binding **(G, H)**.

In order to demonstrate that the assay could distinguish AMG 966 from an endogenous antibody, control serum spiked with AMG 966, as well as day 3 serum samples from cohort 2 were utilized. Day 3 was selected because the AMG 966 concentration was relatively high at this time point, and ADA would not interfere with detection of AMG 966. For both AMG 966 spiked and day 3 serum, binding to the TL1A and TNFα flow cell surfaces was confirmed with both the anti-human IgG and 1A3 reagents confirming the presence of drug (**Figures 8A, C, E, G**; AMG 966 spiked control not shown). ADA samples from Cohorts 2 and 7 were chosen to be analyzed in this assay since both were dosed at 70 mg, enabling comparison of single versus multiple dosing regimens, while minimizing the chance that drug would interfere in the assay. When these samples were tested, the TL1A surface showed minimal, transient evidence of endogenous anti-TL1A antibody in cohort 2 (**Figures 8A, C**), and AMG 966 was detected in 1 subject from cohort 7 (**Figures 8B, D**). However, on the TNFα flow cell surface, all dosed subjects in cohort 7 showed increasing signal compared to baseline which was confirmed with the anti-human IgG reagent, but not the AMG 966-specific antibody 1A3, indicating these subjects developed endogenous anti-TNFα antibodies as a result of AMG 966 treatment (**Figures 8F, H**). Binding in dosed subjects from cohort 2 was less pronounced but exhibited a similar trend, as would be expected after a single dose (**Figures 8E, G**). Placebo treated subjects did not exhibit any binding.

## Discussion

Immune complex-mediated immunogenicity was first described in the 1960s. This phenomenon is dependent on the ratio of antibody to antigen and tends to occur in conditions of antigen excess (23). While this mechanism is often considered in the context of vaccine development (24), it generally is not considered during development of a biologic drug. However, as the development of multi-specific protein therapeutics becomes more widespread, the potential for immune complex formation and consequent immunogenicity must be considered, as the latter can lead to both loss of pharmacodynamic activity and safety concerns.

There are several examples of approved therapeutics with potentially immune complex-mediated immunogenicity. For instance, adalimumab and infliximab both form large complexes with targets of 4,000 and 14,000 kDa, respectively (17) and both are immunogenic (25–27). If the complex forming ability of adalimumab is enhanced by adding an IL-17A binding domain, as is the case with ABT-122 or JNJ-61178104 (formerly COVA-322), then the immunogenicity incidence in healthy subjects jumps to 99% and 100%, respectively (28, 29). This phenomenon is not entirely limited to TNFα inhibitors. Pfizer recently reported an 82% incidence of ADA for PF-06480605, a TL1A inhibitor being developed for UC (30).

On the other hand, TNFα inhibitors that bind trimer in a 1:1 ratio, such as etanercept, tend to be much less immunogenic (Etanercept USPI). Furthermore, not all monoclonal antibodies that bind multimeric targets are necessarily immunogenic. An example of this is denosumab, which binds the trimeric target RANKL, but forms drug to target complexes at a limited ratio of 3:2 (31). For a multimeric target, the precise epitope bound by a targeting protein likely plays a significant role in determining immune complex size, and consequently in determining immunogenic risk.

While the aim of this study was not to prove the mechanism of AMG 966 immunogenicity, understanding the nature of immunogenicity from clinical data is important for further engineering of such biologics. The data presented demonstrate a restoration of FcγR binding to the aglycosylated Fc domain as a result of immune complex formation, consistent with previous observations for Fc silenced constructs (20). This partially restored binding activity and could plausibly lead to enhanced antigen uptake and immune cell activation resulting in a loss of tolerance to AMG 966 and, to some extent, TNFα. The endogenous antibody response to TNFα, but not TL1A, suggests that there may be stronger tolerance to TL1A relative to TNFα. This is supported by evidence that antibodies to TNFα exist to some extent even in healthy subjects (32–34) and in individuals with autoimmune disease (35), while a similar literature does not exist for antibodies to TL1A. There is evidence that endogenous antibodies to TNFα may be important in control of disease activity in systemic lupus erythematosus (35), raising the possibility that AMG 966 may have value as a therapeutic vaccine.

Although the totality of the data supports the immune complex hypothesis described above, we cannot rule out an alternative or supplemental mechanism, such as binding of AMG 966 to TL1A and/or TNFα on the surface of antigen presenting cells. There is evidence to indicate that anti-TNFα antibodies that bind transmembrane TNF are effectively processed and presented on MHC class II (36), potentially promoting immunity. Alternatively, it is plausible that an initial T cell-independent response is triggered by the exposed, repeating Fc domains on the outside of the immune complex, consistent with our observation that the early antibody response is largely Fc-specific. An early T cell-independent anti-AMG 966 antibody response could restore the ability of the AMG 966-target immune complex to bind FcγR and complement, since these endogenous antibodies would have normal effector function. Furthermore, while the charge pair mutations initially appeared to be a probable cause for the high incidence of AMG 966 immunogenicity, none of the experiments performed support this hypothesis. The mutations did not create new T cell epitopes and B cell specificity for the mutations was not observed until late in the immune response.

The implications of this study are twofold. First, as multi-specific proteins are advanced into clinical studies, the ability to form immune complexes with soluble target should be thoroughly assessed *in vitro* as part of the immunogenic risk assessment using tools such as SEC-MALS. Sequence-based risk assessments such as T cell assays are necessary, but not sufficient, and it is critical that the pharmacology of the drug is considered when assessing the immunogenic risk. Second, it’s possible that patients being treated with other immune complex forming TNFα inhibitors may benefit from the treatment, even in the context of a robust ADA response, due to an endogenous anti-TNFα antibody response. This phenomenon could also explain why TNFα inhibitors show differential efficacy in certain diseases. A clinical assay to measure endogenous anti-TNFα antibodies may be a valuable tool for physicians to further understand a patient’s response to a TNFα inhibitor and make prudent treatment decisions.

## Data Availability Statement

Qualified researchers may request data from Amgen clinical studies. Complete details are available at the following: https://wwwext.amgen.com/science/clinical-trials/clinical-data-transparency-practices/clinical-trial-data-sharing-request/. Requests to access the datasets should be directed to https://wwwext.amgen.com/science/clinical-trials/clinical-data-transparency-practices/clinical-trial-data-sharing-request.

## Ethics Statement

The study protocol was approved by Quorum Review IRB. Written informed consent was obtained from each study participant prior to participating in the study. The study was conducted in accordance with the ethical principles of the Declaration of Helsinki and International Council for Harmonisation Good Clinical Practice guidelines.

## Author Contributions

MK, TB, JH, MM, LH, KK, RP, and SK designed and carried out experiments. MK, HH, YB, VF-SC, MT, BS, LC, JP, and DM designed the clinical study and performed data analysis. MK wrote the manuscript. All authors contributed to the article and approved the submitted version.

## Funding

This research was funded by Amgen.

## Conflict of Interest

All authors were employees of Amgen during the time the study was being conducted.

The authors declare this study received funding from Amgen. The funder was responsible for study design, interpretation of data, and the writing of this article. AMG 966 is disclosed in patent application WO2017/106383.

## Publisher’s Note

All claims expressed in this article are solely those of the authors and do not necessarily represent those of their affiliated organizations, or those of the publisher, the editors and the reviewers. Any product that may be evaluated in this article, or claim that may be made by its manufacturer, is not guaranteed or endorsed by the publisher.
