# Supplementary material for: Immune Complex Formation Is Associated With Loss of Tolerance and an Antibody Response to Both Drug and Target
Source: Front Immunol. 2021 Dec 14;12:782788. doi: 10.3389/fimmu.2021.782788 (PMC8712722; doi:10.3389/fimmu.2021.782788)
Supplement: Supplementary file 1 [file DataSheet_1.pdf]

Supplementary Material

1 Supplementary Tables

Supplementary Table 1. Novel agretopes created by charge pair mutations based on IEDB analysis using ≤1% percentile rank threshold

TNFα heavy chain			TNFα light chain		
**Predicted agretope**	**Allele**	**Percentile rank**	**Predicted agretope**	**Allele**	**Percentile rank**
**YSLKSVVTVPSSSLG**	DRB1*08:02	0.38	DSTYSLESTLTLSKA	DRB1*04:01	1.00
**LYSLKSVVTVPSSSL**	DPA1*02:01/DPB1*14:01	0.49			
**GLYSLKSVVTVPSSS**	DRB1*08:02	0.52			
**LYSLKSVVTVPSSSL**	DRB1*08:02	0.52			
**SLKSVVTVPSSSLGT**	DRB1*08:02	0.55			
**YSLKSVVTVPSSSLG**	DPA1*02:01/DPB1*14:01	0.65			
**GLYSLKSVVTVPSSS**	DPA1*02:01/DPB1*14:01	0.92			

Supplementary Table 2. Relative potency of FcγR binding at various ratios of AMG 966, TNFα, and TL1A

Sample ratio AMG 966:TNFα:TL1A	FcγRIa % Rel Pot	FcγRIIa % Rel Pot	FcγRIIIa % Rel Pot
1:0:0 (AMG 966 alone)	0.0	0.6	0.0
1:1:1	Undetermined	0.0	0.7
4:1:1	Undetermined	11.5	0.2
9:1:1	Undetermined	10.2	0.1
100:1:1	0.1	Not tested	Not tested

2 Supplementary Figure Legends

Supplementary Figure 1. AMG 966 is a heteroimmunoglobulin molecule, capable of neutralizing both TNFα and TL1A. The approximate locations of positive and negative charge pair mutations are shown as red and black circles, respectively. Lysine was used to provide the positive charge and aspartic or glutamic acid for negatively charged residues. The Fc domain is stable effector functionless.

Supplementary Figure 2. AMG 966 first-in-human trial study schema. The study consisted of 6 single ascending dose cohorts (**A**) and 3 multiple ascending dose cohorts with Q2W dosing (**B**).

Supplementary Figure 3. Pharmacodynamic assessments of serum TNFα and TL1A. Serum TL1A was measured for all subjects in the SAD (**A**) and MAD (**B**) cohorts throughout the course of the study and trended back to baseline over time. (**C**) TNFα in the MAD cohorts showed a similar trend as the SAD.
