# Supplementary figures and images for: Immune Complex Formation Is Associated With Loss of Tolerance and an Antibody Response to Both Drug and Target
Source: Front Immunol. 2021 Dec 14;12:782788. doi: 10.3389/fimmu.2021.782788 (PMC8712722; doi:10.3389/fimmu.2021.782788)

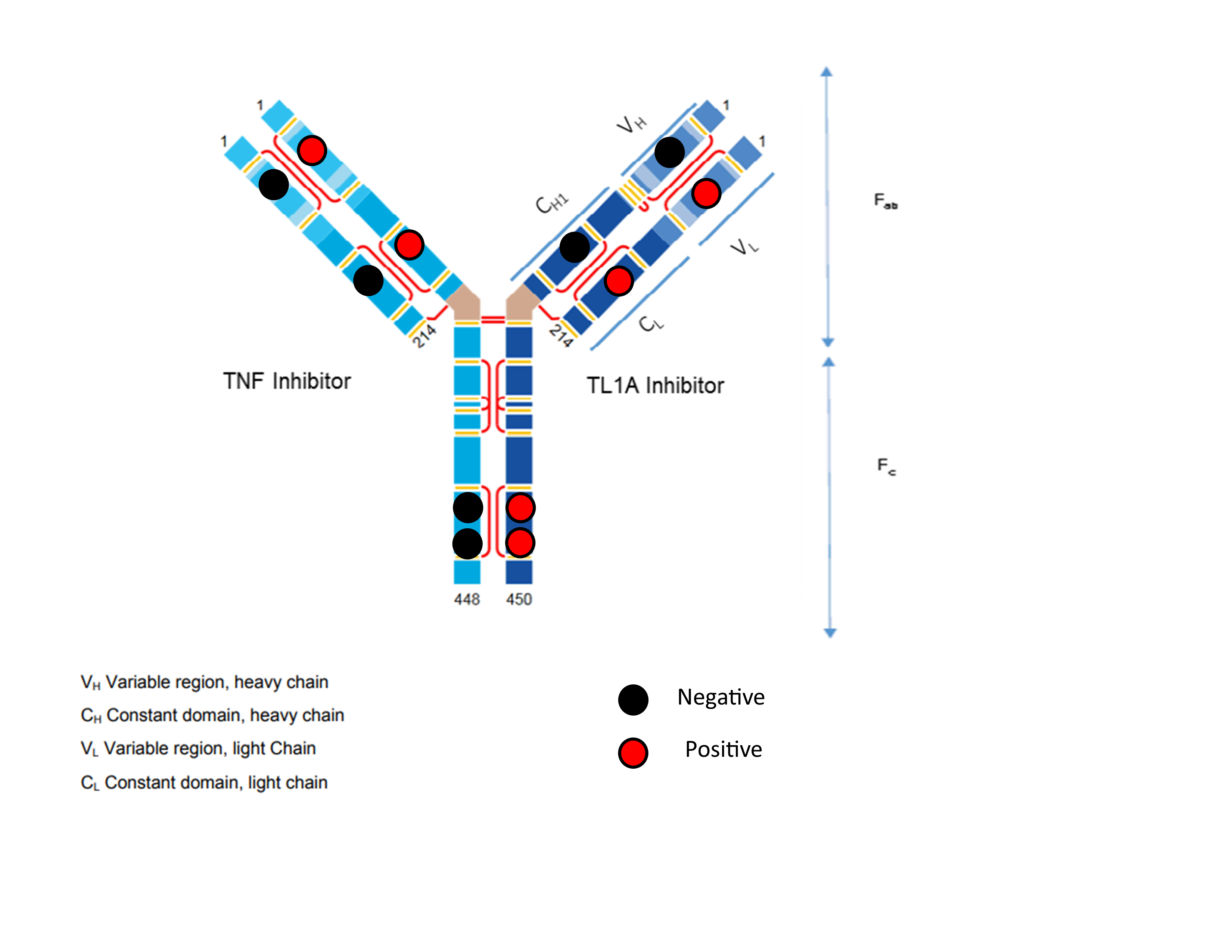

Supplement: Supplementary file 2 [file Image_1.tif]

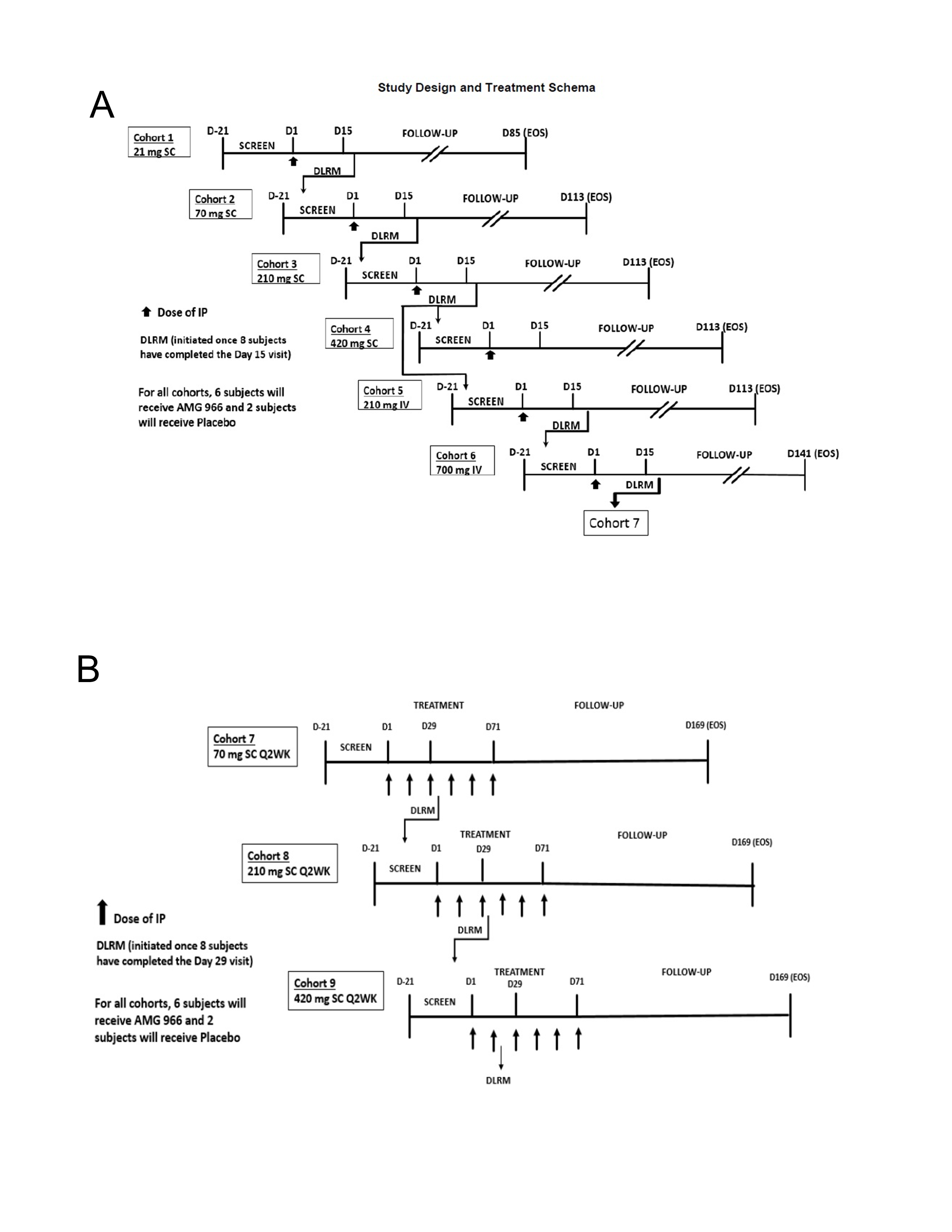

Supplement: Supplementary file 3 [file Image_2.tif]

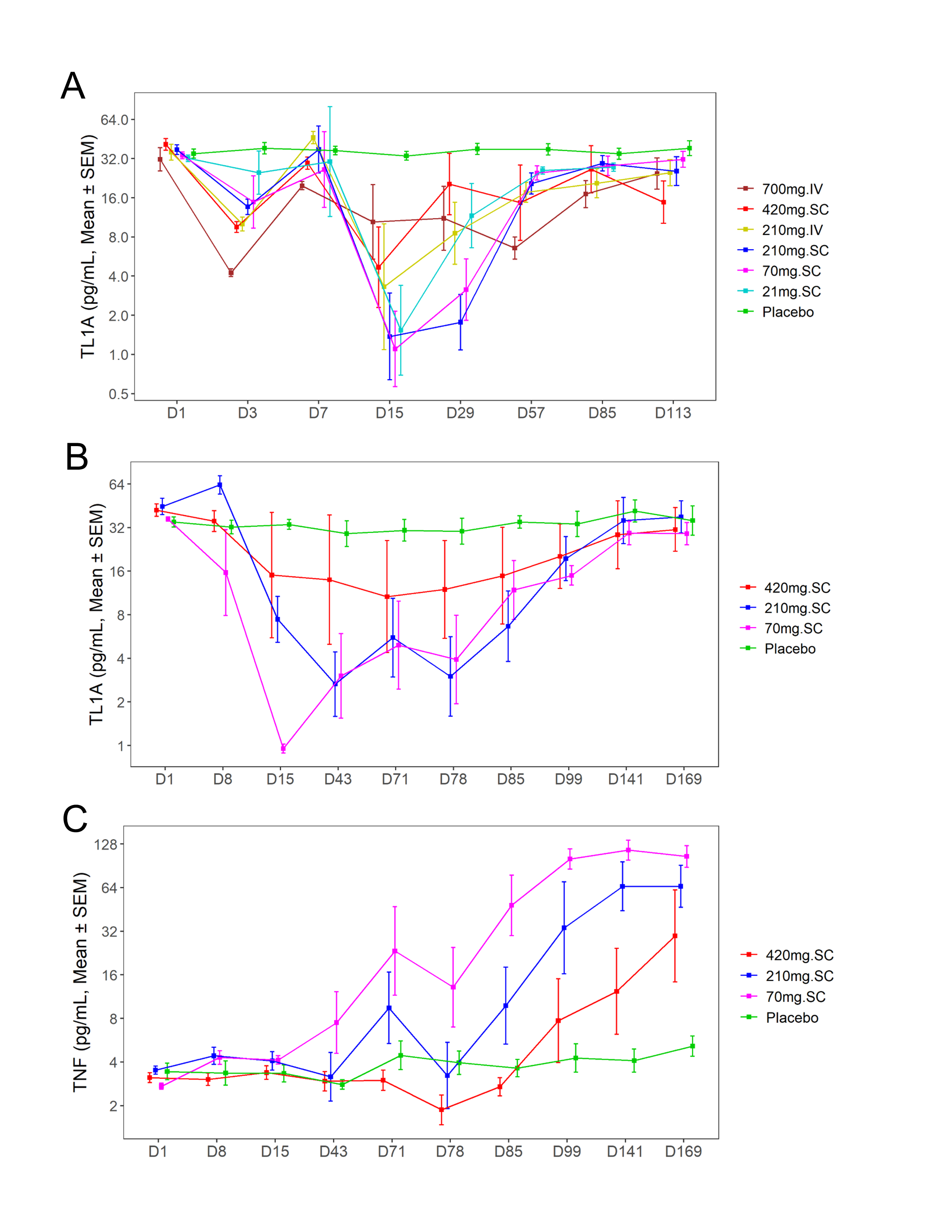

Supplement: Supplementary file 4 [file Image_3.tif]
